# Follicle-stimulating hormone signaling in Sertoli cells: a licence to the early stages of spermatogenesis

**DOI:** 10.1186/s12958-022-00971-w

**Published:** 2022-07-02

**Authors:** Jia-Ming Wang, Zhen-Fang Li, Wan-Xi Yang, Fu-Qing Tan

**Affiliations:** 1 The Sperm Laboratory, College of Life Sciences, Zhejiang University, Hangzhou, 310058 China; 2 The First Affiliated Hospital, College of Medicine, Zhejiang University, Hangzhou, Zhejiang 310003 China

**Keywords:** Follicle-stimulating hormone, Sertoli cell, Signaling pathway, Spermatogenesis, FSH treatment

## Abstract

Follicle-stimulating hormone signaling is essential for the initiation and early stages of spermatogenesis. Follicle-stimulating hormone receptor is exclusively expressed in Sertoli cells. As the only type of somatic cell in the seminiferous tubule, Sertoli cells regulate spermatogenesis not only by controlling their own number and function but also through paracrine actions to nourish germ cells surrounded by Sertoli cells. After follicle-stimulating hormone binds to its receptor and activates the follicle-stimulating hormone signaling pathway, follicle-stimulating hormone signaling will establish a normal Sertoli cell number and promote their differentiation. Spermatogonia pool maintenance, spermatogonia differentiation and their entry into meiosis are also positively regulated by follicle-stimulating hormone signaling. In addition, follicle-stimulating hormone signaling regulates germ cell survival and limits their apoptosis. Our review summarizes the aforementioned functions of follicle-stimulating hormone signaling in Sertoli cells. We also describe the clinical potential of follicle-stimulating hormone treatment in male patients with infertility. Furthermore, our review may be helpful for developing better therapies for treating patients with dysfunctional follicle-stimulating hormone signaling in Sertoli cells.

## Background

Spermatogenesis is a process that is under complex regulation to achieve successive germ cell proliferation and differentiation [1]. Starting from spermatogonia stem cells (SSCs) producing differentiated spermatogonia, differentiated spermatogonia transform into spermatocytes. Spermatocytes undergo meiosis to produce round spermatids in which the chromosome number is reduced from diploid to haploid [2]. Round spermatids then undergo transformation to form the final spermatozoa which are released into the lumen. In mammals, only Sertoli cells and undifferentiated spermatogonia are detected during prepubertal and juvenile periods while spermatogenesis is initiated at puberty when undifferentiated spermatogonia begin to differentiate and enter meiosis [3–5].

As the only type of somatic cell in seminiferous tubule, Sertoli cell (SC) functions as a ‘nurse’ to care for spermatogenesis via paracrine actions to provide necessary nutrition and factors as well as forming necessary structures such as the blood-testis barrier (BTB) and Sertoli cell-Germ cell adhesion complex [6–9]. In higher vertebrates, spermatogenesis requires hormonal regulation by the hypothalamic-pituitary gonadal axis [10, 11]. Gonadotropin-releasing hormone (GnRH) is synthesized in the hypothalamus and is released into the pituitary gland where it stimulates the secretion of two gonadotropins, follicle-stimulating hormone (FSH) and luteinizing hormone (LH) [12]. FSH and LH then enter the circulation system to act on testis. Only undifferentiated spermatogonia and Sertoli cells are present in seminiferous tubule in the absence of FSH and LH [13]. Hormonal regulation of spermatogenesis is important and is mediated indirectly by SCs. Here, we will focus on the function of FSH signaling in spermatogenesis.

FSH is a glycoprotein that plays an essential role in prepubertal preparation for spermatogenesis and pubertal spermatogenesis regulation [14]. Its receptor FSH receptor (FSHR) is exclusively expressed on the cellular membrane of Sertoli cells [15]. In early life of both primates and rodents, physiological role of FSH signaling in spermatogenesis is to stimulate the transcription of genes related to DNA replication and cell cycle progression [16, 17]. Decades of study using the hypogonadal (*hpg*) model [18], FSHβ subunit knockout model [19], GnRH-immunized model [20] and FSHR knockout model [21–23] have revealed pivotal roles for FSH in regulating Sertoli cell function, increasing spermatogonia number, promoting entry into meiosis and limiting overall germ cell apoptosis. Since adult FSHR knockout mice are fertile but exhibit a reduced sperm output and completion of meiosis mainly depends on testosterone action, FSH is suggested to play a dominant role in establishing the most important parameter for testicular development and spermatogenesis prior to puberty in rodents [21, 24–27]. In men, FSH is essential to maintain fertility. Subfertility with quantitatively reduced spermatogenesis will occur in the absence of FSHR function [28] while a mutation in FSHβ subunit leads to azoospermia and infertility [29]. Although studies have provided a better understanding of the spermatogenesis processes that are regulated by FSH signaling, few molecules participating in these regulating activities have been precisely identified. We are also unable to elucidate the exact role of FSH in human spermatogenesis [30]. As a result, a review of the present work about FSH signaling in SCs is necessary and suggestions for future studies should be proposed. Moreover, FSH treatment has the potential to improve sperm number and motility in patients with hypogonadotropic hypogonadism or normogonadotropic patients with idiopathic impairment of spermatogenesis, highlighting the importance of obtaining a better understanding of FSH signaling in humans [31–34]. All of our hard work aims to achieve the ‘bench to bedside’ translation and cure more patients with FSH signaling dysfunction.

We surveyed articles in PubMed using the following keywords: ‘Sertoli cell’, ‘FSH’, ‘spermatogenesis’, ‘Sertoli cell proliferation’, ‘Sertoli cell differentiation’, ‘Spermatogonia stem cell self-renewal’, ‘meiosis’, ‘Spermatogonia proliferation’, ‘apoptosis’, ‘hypogonadotropic hypogonadism’, ‘normogonadotropic’, ‘FSH treatment’. We will present this review at the cellular and molecular levels, covering four parts: 1) Sertoli cell proliferation, differentiation and apoptosis; 2) Spermatogonia pool maintenance, differentiation and spermatogonia survival; 3) Entry into meiosis and spermatocyte survival; 4) Potential use of follicle-stimulating hormone in treating male infertility. Experimental species include rats, mice, zebrafish, sheep, bovines, goats, newts, trout and men. Our review is focused on the function of FSH signaling in SCs during the early stages of spermatogenesis.

## Overview of follicle-stimulating hormone signaling in Sertoli cell

FSH is a glycoprotein composed of ɑ and β subunits. FSHɑ is a subunit shared with other glycoproteins, while FSHβ subunit is unique to FSH [35]. FSH exerts its function through the interaction between FSHβ and FSHR [14, 36]. According to a recent analysis of crystal structure, FSHR, which is a heterotrimeric guanine nucleotide–binding proteins (G proteins)-coupled receptor, is composed of a hormone binding domain, hinge region, hairpin loop, seven-transmembrane α helical domains and an intracellular domain [37–39]. The binding of FSH to the FSHR hormone-binding domain leads to a conformational change in FSHR, which facilitates the interaction between residues of FSHɑ and FSHβ subunit with the residues of the hinge region of FSHR. This interaction will further alter the conformation of seven-transmembrane α helical domains, resulting in the transmission of the signal to the intracellular domain, where coupling to effectors, recruiting adaptor proteins and transmitting FSH signaling downstream happen [39–41]. For more crystal structures, please see a review [39].

FSHR is present in the testis before a significant concentration of hormone appears in the foetal circulation [42]. In both rodents and primates, FSHR expression begins in the second half of gestation [43]. The interaction between FSH and FSHR is important for the function of FSH signaling. The variation of FSH/FSHR interaction at different age depends on the amount of SCs expressing FSHR with respect to those not expressing FSHR. In mice, FSH binding peaks between Days 7 and 21 but decreases significantly between Days 20 and 37 [44]. In rats, the *Fshr* mRNA level increases until Day 7, remains constant for 10 days and then decreases sharply on Day 40 [14]. The initial increase correlates with the proliferation of SCs and the increase of FSHR density per SC. While the sharp decrease correlates with the wide appearance of spermatocytes and spermatids so that the ratio of SCs to germ cells drops per seminiferous tubule. During one cycle of spermatogenesis in rats, FSH binding and *Fshr* mRNA level peak in stages XIII, XIV and I during the early development of germ cells but reach their lowest levels in stages VII and VIII when germ cells have developed to mature state [45–47]. Based on these observations, it can be concluded that FSH signaling mainly participates in the initiation and early development phases of spermatogenesis.

**Fig. 1:**
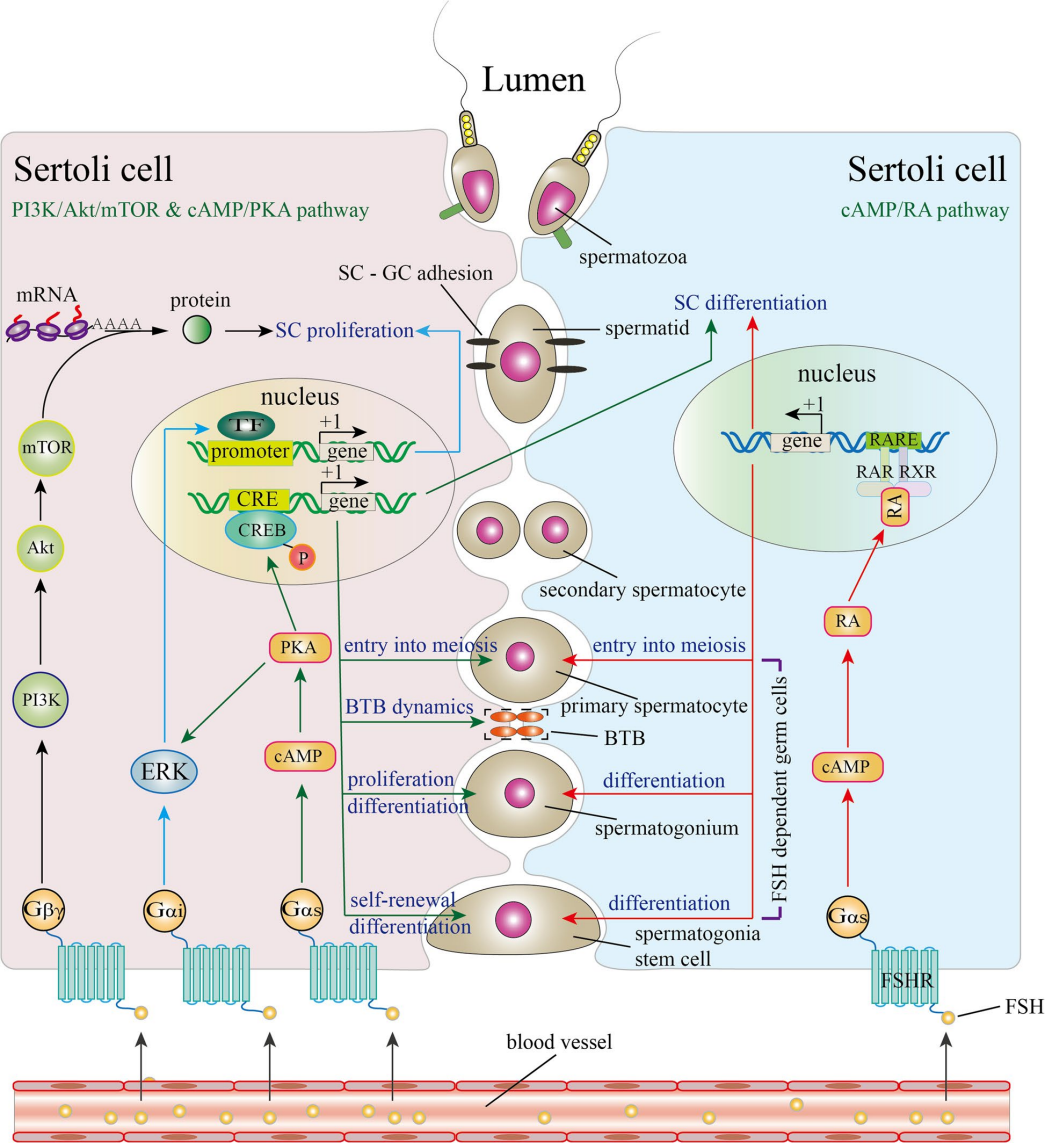
FSH signaling pathway in SCs. After FSH binds to FSHR on the membrane of SCs, FSH signaling is activated. FSHR recruits different types of G proteins to mediate different signaling pathways. Recruitment of Gβγ subunits activates the PI3K/Akt/mTOR signaling pathway, promoting the translation of mRNAs. Recruitment of the Gɑ subunit activates the cAMP/PKA signaling pathway. Activated PKA directly phosphorylates the CREB protein in the nucleus. Phosphorylated CREB binds to CREs of target genes to regulate transcriptional activity. In addition, PKA activates ERK during Sertoli cell proliferation. ERK activation is also mediated by recruiting the Gɑi subunit. Recently, FSH-induced RA signaling was reported. FSH promotes RA biosynthesis through a cAMP-dependent pathway. RA translocates into the nucleus and binds to RAR/RXR to regulate target gene transcription. The cAMP/PKA signaling pathway participates in Sertoli cell differentiation, SSC self-renewal and differentiation, spermatogonia proliferation and their entry into meiosis, as well as BTB dynamics. The cAMP/PKA/ERK signaling pathway and PI3K/Akt/mTOR signaling pathway induce Sertoli cell proliferation. The cAMP/RA signaling pathway has been shown to participate in SSC differentiation, spermatogonia differentiation and their entry into meiosis. TF: transcription factor

To date, at least five FSH signaling pathways have been identified in SCs: cyclic adenosine monophosphate (cAMP)/protein kinase A (PKA) pathway, extracellular-regulated kinase (ERK)/mitogen-activated protein kinase (MAPK) pathway, phosphoinositide 3-kinase (PI3K) pathway, calcium pathway and phospholipase A2 pathway. Here we mainly review the first three types of these five types pathways as well as the newly found retinoid acid pathway. Only these four types of pathways are included in Fig. 1. The cAMP/ PKA signaling pathway was the first to be identified. Upon FSH binding to FSHR on the plasma membrane of SCs, FSHR couples to the Gɑs subunit to activate adenylate cyclase (AC). Activated AC recruits ATP and transforms it into cAMP [48]. cAMP then binds to the regulatory subunits of PKA to release catalytic subunits of PKA [49, 50]. Catalytic subunits translocate into the nucleus and phosphorylate cyclic AMP response-element binding protein (CREB) at Ser133 or some cAMP-responsive elemental modulators [14]. These factors bind to the cAMP-response element of target genes to regulate their transcriptional activity during spermatogenesis [51]. Additionally, FSH activates ERK/MAPK pathway by coupling to both the Gɑi and Gɑs subunits in vitro [52]. The interaction of FSHR with the Gɑs subunit leads to ERK activation via a cAMP/PKA dependent pathway while the exact pathway mediated by FSHR coupling to Gɑi remains to be determined. Moreover, coupling of FSHR to Gβγ also activates PI3K [53, 54]. Activated PI3K triggers the transition from phosphatidylinositol bisphosphate (PIP2) to phosphatidylinositol 3,4,5-trisphosphate (PIP3) [55]. Accumulation of PIP3 leads to the phosphorylation of protein kinase B (Akt) and mammalian/mechanistic target of rapamycin (mTOR) [53, 56–59]. Activated mTOR phosphorylates 70-kDa ribosomal S6 kinase (p70S6K) to promote protein translation and gene expression [53, 60, 61]. Beta-arrestin promotes the internalization of FSHR to sustain the prolonged activation of signaling. This internalization is mediated by clathrin proteins [31, 62]. Interestingly, the levels and biochemical characteristics of signaling messengers are stage-specific upon FSH stimulation. The FSH-induced cAMP production level increases from birth to puberty while the FSH-induced PIP3 production level decreases from birth to puberty. Additionally, the p70S6K phosphorylation sites differ between different developmental periods. p70S6K is phosphorylated at T389, T421 and S424 via the cAMP/PKA pathway and PI3K/Akt pathway during proliferating stage but is only phosphorylated at T389 by the cAMP/PKA pathway during differentiating state [53]. The signaling pathway adopted is also stage-specific. For example, FSH mediated ERK activation in vitro was only detected in 5 and 11 day old rats, not in 19 day-old rats. Recently, FSH signaling was linked to retinoic acid (RA) signaling in SCs. FSH stimulates RA synthesis during the postnatal period via cAMP-dependent upregulation of retinol dehydrogenase 10 (RDH10) and aldehyde dehydrogenase 1A1 (ALDH1A1) [63–65]. During the pubertal period, FSH facilitates the translocation of retinoic acid receptor ɑ (RARɑ) into the nucleus. With the help of cytoplasmic RA-binding protein 2 (CRABP2), RA interacts with the RAR/retinoid X receptor (RXR) heterodimer and binds to RA response elements (RAREs) to regulate gene transcription [66, 67]. Other signaling pathways, including the calcium pathway and phospholipase A2 pathway have been reviewed by other researchers (For reviews, please see [68–70]).

## Follicle-stimulating hormone signaling in Sertoli cell regulates early stages of spermatogenesis

### Roles of FSH mediated signaling in Sertoli cell proliferation, differentiation and apoptosis

**Fig. 2:**
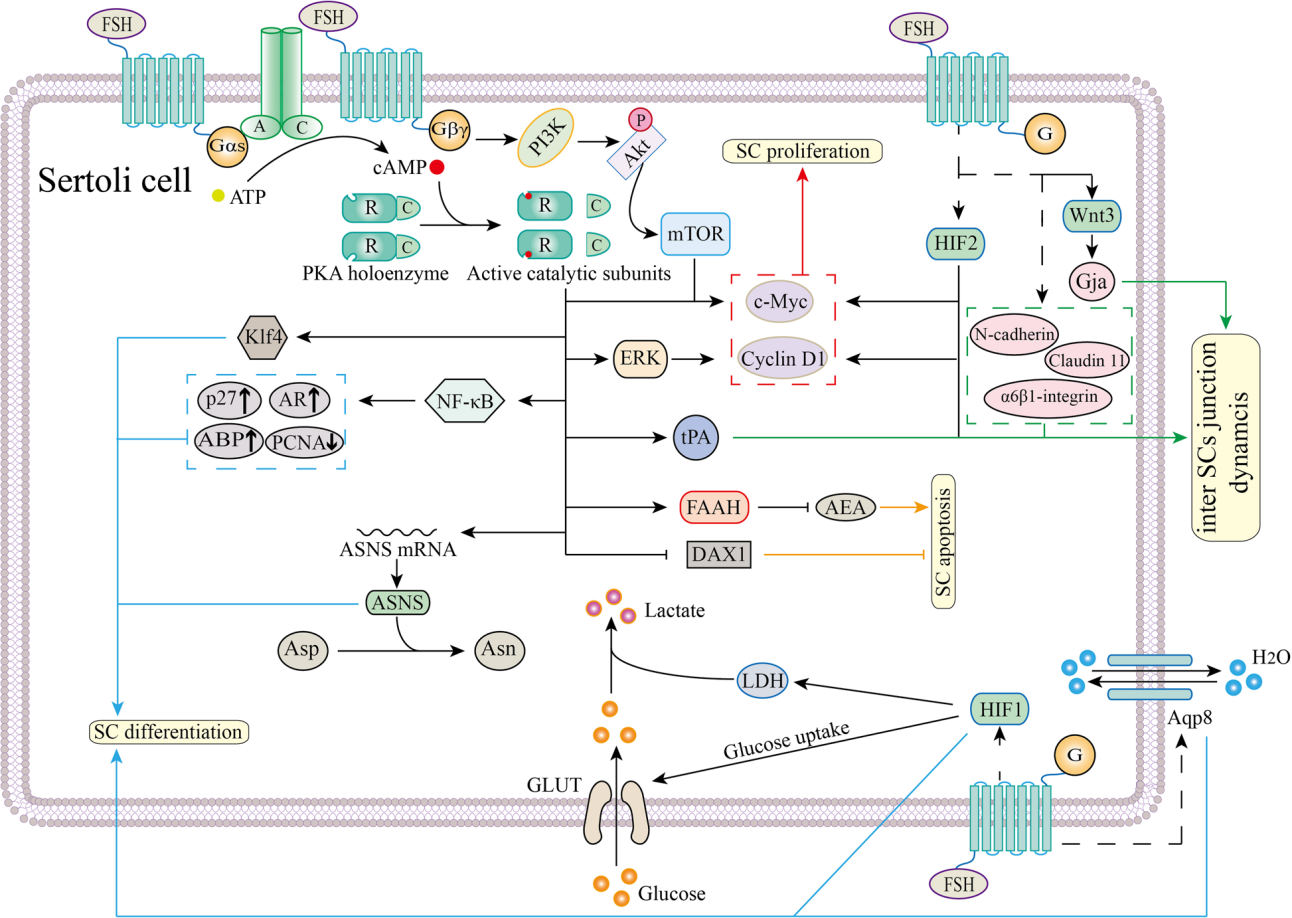
Functions of FSH signaling in SC proliferation, differentiation and apoptosis. Binding of FSH to FSHR recruits Gɑs to FSHR, activating AC. AC transforms ATP into cAMP, and cAMP binds to regulatory subunits of PKA to release catalytic subunits of PKA. Activated PKA induces the expression of c-Myc and Cyclin D1 to promote SC proliferation (red). c-Myc expression is also upregulated by the PI3K/Akt/mTOR pathway. The cAMP/PKA/ERK pathway leads to the expression of Cyclin D1 only during SC proliferation. Through an unknown pathway, FSH induces the expression of HIF2 to promote c-Myc and Cyclin D1 expression. Moreover, inter-SC junction dynamics are also mediated by FSH signaling (green). FSH induces tPA expression via a cAMP/PKA-dependent pathway and three components of the BTB, N-cadherin, ɑ6β1-integrin and claudin 11, through an unknown pathway. Combined with testosterone, FSH stimulates Gja expression via the Wnt3 pathway. Additionally, FSH participates in SC differentiation (blue). Klf4, NF-κB and ASNS expression are mediated by the cAMP/PKA signaling pathway. HIF1 and Aqp8 expression are mediated by an unknown pathway. HIF1 mainly participates in the glycolytic pathway, and Aqp8 is important for water balance. Finally, the cAMP/PKA-mediated balance of FAAH and DAX1 maintains normal SC apoptosis (orange). The dotted line represents an unknown mechanism. For more information, please see the main text

SCs create a microenvironment and provide necessary nutrition for germ cells to complete spermatogenesis. The final number of SCs in adulthood is determined by the proliferation activity during the prepubertal period. SC differentiation during puberty endows SCs with their functions in spermatogenesis. Additionally, SC apoptosis maintains a healthy SC pool. All three processes are regulated by FSH signaling (Fig. 2).

#### Sertoli cell proliferation

The final number of SCs determines the quality and quantity of spermatogenesis. SC proliferation occurs in the foetal or neonatal period and in the peripubertal period in all species [16]. Decades of studies using decapitation model, FSH antagonist model, transgenic model and FSHR knockdown model have revealed that FSH signaling in SCs is essential for SC proliferation.

FSH regulates SC proliferation only during foetal and early postnatal life. Pioneering works using the [^3^H] -thymidine assay, which indicates mitosis activation, found that low levels of endogenous FSH decrease [^3^H]-thymidine incorporation and the final SC number both in vivo and in vitro [71–73]. Injection of human FSH into immature rats with FSH withdrawal restored the mitotic activation and the final SC number [74–77]. In addition, treating *hpg* mice with recombinant FSH or expressing an FSH transgene in *hpg* mice also counteracted the negative effect of FSH deprivation on SC proliferation. FSHR mutation also decreased the SC number in mice, supporting these results [21, 22, 78].

Molecular mechanism underlying this stimulatory effect has been elucidated (Table 1). The main pathway that is included in this period is the PI3K/Akt signaling pathway [69, 79]. PI3K/Akt pathway phosphorylates p70S6K at T389, T421 and S424 [53]. Furthermore, Riera et al. reported that FSH also regulates SC proliferation via the PI3K/Akt/mammalian target of rapamycin complex 1(mTORC1) pathway. Supporting these results, phosphorylated Akt, phosphorylated proline-rich Akt substrate of 40 kDa (PRAS40), phosphorylated mTOR and phosphorylated p70S6K were detected after FSH stimulation in vitro [80]. Moreover, Crépieux et al. showed that FSH supports cAMP/PKA dependent extracellular signal-regulated protein kinases 1 and 2 (ERK1/2) activation and subsequent activation of the MAPK cascade in vitro [52].

**Table 1:** Factors that are under FSH signaling regulation during early stages of spermatogenesis

Process	Molecules	Signaling pathway	References
Sertoli cell proliferation	c-Myc	cAMP/PKA & PI3K/Akt/mTORC1	[80–82]
	Cyclin D1	cAMP/PKA/ERK	[83]
	HIF2	unknown	[84–86]
Sertoli cell differentiation	Klf4	cAMP/PKA	[87, 88]
	NF-κB	unknown	[89]
	HIF1/2	unknown	[90]
	c-jun, jun-B	unknown	[91]
	tPA	cAMP/PKA	[92, 93]
	ASNS	cAMP/PKA	[94]
	Aqp8	unknown	[95, 96]
	Gja	FSH/Wnt3	[97]
	N-cadherin, ɑ6β1-integrin	unknown	[98–100]
	PFKFB1/3	unknown	[101]
	PDK3	unknown	[101]
Sertoli cell apoptosis	FAAH	cAMP/PKA	[102]
	DAX1	cAMP/PKA	[103]
Spermatogonia maintenance	GDNF	cAMP/PKA	[104, 105]
	FGF2	cAMP/PKA	[106, 107]
	PGE2	unknown	[108]
Spermatogonia stem cell differentiation	BMP4	cAMP/PKA	[109, 110]
	SCF, SLF	cAMP/PKA	[111, 112]
	Igf3	cAMP/PKA	[113, 114]
	transferrin	unknown	[115, 116]
Spermatogonia survival	Bok	cAMP/PKA	[117, 118]
Entry into meiosis	Activin A, Inhibin B	cAMP/PKA	[119, 120]
	IL-6	cAMP/PKA & cAMP/PKC	[121]
	nociceptin	cAMP/PKA	[122, 123]
	Nrg1, Nrg3	unknown	[124, 125]
Spermatocyte survival	Gal-3	cAMP/PKA	[126, 127]
	AP-1	unknown	[128]

Furthermore, genes regulated by FSH signaling that promote SC proliferation have been identified. Most genes are related to DNA replication, the cell cycle, cytoskeletal rearrangement and stem cell factors. Among them, cell-derived Myc (c-Myc) and type D1 cyclin (Cyclin D1) have been linked to the FSH signaling pathway. The proto-oncogene *c-myc* encodes the transcription factor c-Myc, which is important for promoting cell growth and maintaining vitality [81]. In prepubertal rats, the expression of *c-myc* mRNA was elevated by FSH stimulation via a cAMP-dependent pathway [82]. Further study using rat SCs found that PI3K/Akt/mTOR signaling participate in FSH stimulation of c-myc expression [80]. Cyclin D1, a member of cyclin, binds to cyclin-dependent kinase 4 and 6 to form a complex that promotes cell cycle progression from G1 to S phase [83]. By activating the cAMP-dependent ERK pathway in rat SCs, FSH stimulates Cyclin D1 expression in neonatal rat testes to promote SC proliferation [52]. Hypoxia inducible factor (HIF) is a transcription factor that regulates cell metabolism [84–86]. HIF1 regulates the expression of genes in the glycolytic pathway, while HIF2 regulates the expression of genes related to cell cycle progression [129–132]. During rat SC proliferation, FSH only upregulates HIF2 expression to increase c-Myc and Cyclin D1 expression both in vivo and in vitro through an unknown pathway [130]. Other genes that are regulated by FSH include hairy/enhancer of split gene 1, max-interacting protein repressor and Nur-related protein 1 in murine SCs [133]. Moreover, FSH signaling also cross-talks with insulin growth factor signaling to promote mouse SC proliferation. It is reported that FSH amplifies insulin growth factor signaling mediated Akt phosphorylation [134]. Interestingly, in female mice, FSH can stimulate granulosa cells proliferation via inducing Octamer-binding transcription factor 4 (OCT4) expression [135]. OCT4 is also found to be expressed in human SCs [136]. Whether FSH signaling can promote human SCs proliferation via OCT4 is proposed to be investigated. The precise signaling pathway regulating target gene expression after FSH binds to FSHR remains to be determined.

#### Sertoli cell differentiation

SC differentiation begins after SC proliferation cessation during puberty in all species [16]. During SC differentiation, SCs form the BTB to separate the adluminal area and basalarea. Also, SCs undergo metabolism to provide nutrition for germ cells between them. FSH is maintained at a relatively high level during this stage and promotes SC differentiation via an absolutely different signaling pathway compared with SC proliferation [1].

The main pathway by which FSH regulates SC differentiation is the cAMP/PKA signaling pathway. Although debates exist regarding whether FSH promotes SC differentiation, some evidences support our hypothesis. Firstly, FSH deprivation or FSHR knockout in mature mouse SCs led to low sperm counts and the SC transition from differentiation to proliferation [137]. Secondly, FSH activates ERK in immature rat SCs but inhibits its activation in mature SCs via cAMP/PKA signaling [52]. Thirdly, p70S6K is only phosphorylated at T389 in mature rat SCs, while p70S6K is phosphorylated at T389, T421 and S424 in proliferating cells [53]. Fourthly, cAMP and stimulatory Ga production in pubertal rat SCs are greater than those in neonatal rat SCs and FSH mediated cAMP signaling increases stimulatory Ga production in pubertal rat SCs [138]. Further support is obtained from evidence that FSH inhibits Yes-associated protein (YAP) expression to inhibit the Ste20-like protein kinase Hippo (Hippo) signaling pathway in pubertal rat SCs [139]. Hippo signaling pathway is known to promote cell proliferation [140]. Additionally, an increase in FSH level during puberty promotes RARa to translocate into the nucleus, which is important for SC differentiation [67]. In summary, FSH mainly regulates the cAMP/PKA signaling pathway to promote SC differentiation.

FSH regulates SC differentiation directly and indirectly via targeting direct functional factors and transcription factors respectively (Table 1). First class is transcription factors. Krüppel-like factor 4 (Klf4) is a pleiotropic zinc finger transcription factor that induces the expression of genes involved in SC differentiation. Klf4 expression is induced via cAMP/PKA signaling pathway in the TM4 Sertoli cell line [87, 88]. A recent in vivo study using mice demonstrates that FSH is able to induce expression of Klf4 via suppressing microRNA-92a-3p [141]. Nuclear factor (NF)-κB, a transcription factor that induces expression of genes related to SC function such as androgen binding protein, androgen receptor, is activated during SC differentiation following FSH stimulation in rats [89]. In rat mature SCs, both HIF1 and HIF2 expression are induced under FSH regulation. HIF1 increases glucose transporter 1 (*Glut1*) mRNA level to augment glucose uptake while HIF2 promotes the expression of tight junction protein ZO-1, ZO-2 and Occludin levels to establish the BTB [90]. Also, in vitro study using rat SCs indicated that FSH inhibits the expression of cell-derived jun proto-oncogene (*c-jun*) and increases *jun-B* mRNA level to regulate the transcription factor activator protein-1 (AP-1). AP-1 participates in the transcription response to hormones and growth factors which are necessary for SC differentiation [91].

In addition to transcription factors, direct functional factors involved in structural establishment, biochemical reactions and cell morphology were identified. Asp synthetase (ASNS), which promotes Asp accumulation in SCs, is regulated by FSH to induce its transcription in rat SCs. FSH activates the cAMP/PKA signaling pathway to regulate ASNS expression and its function in SC metabolism [94]. Besides Asp accumulation, FSH also positively regulates lactate production via glycolysis process in SCs [142]. Through interaction with PI3K, FSH promotes translocation of Glut1 to plasma membrane to absorb more glucose in rat SCs [143]. Also, FSH induces the transcription level of bifunctional enzyme 6-phosphofructo-2-kinase/fructose-2,6-biphosphatase (PFKFB) isoform 1 (PFKFB1) and 3 (PFKFB3) in rat SCs to regulate synthesis and degradation of fructose 2,6-biphosphate [101]. What’s more, FSH can inhibit the transition of pyruvate to acetyl-coA through increasing the expression of pyruvate dehydrogenasekinase 3 (PDK3) in rat SCs [101]. All the efforts are to produce more lactate to nourish germ cells. Aquaporin 8 (Aqp8), which is involved in the water balance of rat and mouse SCs, is also stimulated by FSH [95, 96]. FSH is shown to be permissive for the formation of BTB and Sertoli cell – Germ cell junction such as ectoplasmic specialization and adherent junctions [133, 144]. Neural-cadherin (N- cadherin) and alpha6beta1-integrin (a6β1 integrin), two molecules known to make up of ectoplasmic specialization, are upregulated during the rat Sertoli cell differentiation process. FSH participates in this promotion in vitro, along with claudin-11, which belongs to tight junction protein [98–100]. Combination of FSH and testosterone also regulates the expression of gap junction protein Gja via wingless-type MMTV integration site family, member 3 (Wnt3) pathway in mice [97]. Gap junctions are pivotal for germ cell development [145]. Tissue plasminogen activator (tPA) was found to be induced by cAMP/PKA signaling pathway in rat and bovine SCs [92]. As a protease, tPA degrades tight junction proteins to regulate BTB dynamics [93]. Further studies could focus on the linkage between the transcription factor and molecules directly related to SC differentiation, which is helpful to elucidate the complete signaling network downstream FSHR.

#### Sertoli cell apoptosis

Apoptosis is important for maintaining a healthy microenvironment and cell number. SC apoptosis is also regulated by FSH, through the cAMP/PKA signaling pathway. FSH activates the cAMP/PKA signaling pathway to stimulate the expression of N-arachidonoylethanolamine hydrolase (FAAH) and inhibits SC apoptosis that caused by N-arachidonoylethanolamine (AEA) in mice [102]. AEA initiates apoptosis by inducing DNA fragmentation [146]. Additionally, activation of the cAMP/PKA signaling pathway downregulates nuclear receptor subfamily 0 Group B member 1 (DAX1) in maturing rat SCs [147]. Downregulated DAX1 is associated with a higher number of apoptotic cells [103]. The mechanism by which FSH achieves a balance between apoptosis and survival requires further investigation, but is probably mediated by regulating different transcription factors.

In summary, FSH promotes SC proliferation via the cAMP/PKA/ERK and PI3K/Akt/mTORC1 pathways while regulating SC differentiation and apoptosis via the cAMP/PKA pathway. Through sequential and corporative regulation of these three processes, FSH provides a healthy and functional microenvironment for spermatogenesis. However, the pathways that are activated in different processes and molecule function vary between different experiments. One explanation is that no precise boundaries exist between different stages. At the time of SC differentiation, SC proliferation can also occur. Besides, different species have different developmental stages. The two stages may overlap in some species such as rats [16]. Different cell culture conditions might also explain the difference between the results. In addition, it is suggested to consider whether the autocrine action is involved in SC proliferation and differentiation, as well as whether the autocrine action is regulated by FSH.

### Role of FSH mediated signaling in Spermatogonia pool maintenance, differentiation and survival

**Fig. 3:**
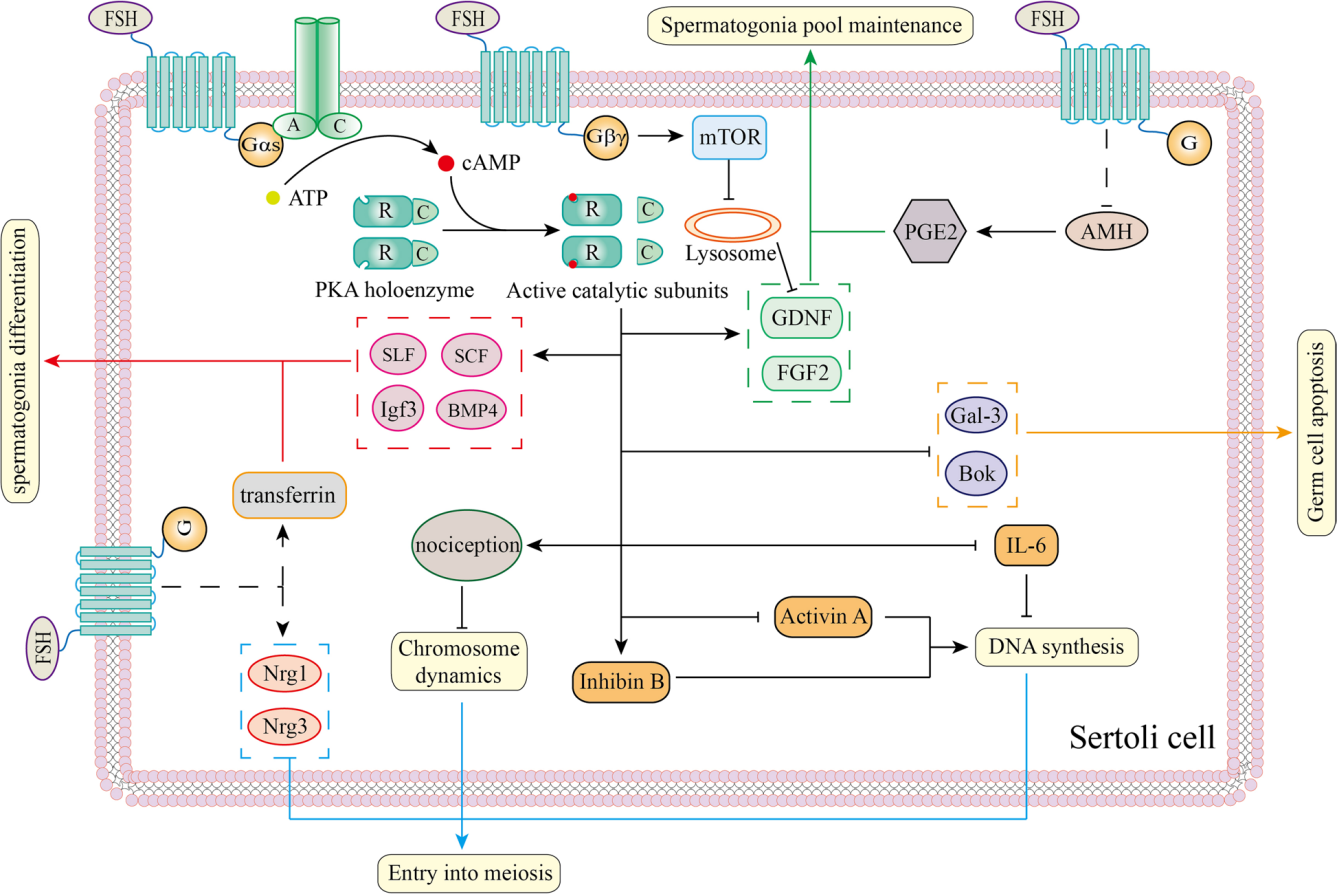
Functions of FSH signaling in early stages of spermatogenesis. Activating cAMP/PKA signaling pathway induces expression of GDNF and FGF2 in SC which will be secreted to SSC to maintain spermatogonia pool. Notably, FSH can inhibit degradation of GDNF via suppressing lysosomal biogenesis. This process is validated by activating PI3K/Akt/mTOR pathway. Additionally, FSH negatively regulates PGE2 through inhibiting AMH. PGE2 is also important for spermatogonia pool maintenance. SLF, SCF, Igf3 and BMP4 that is activated via cAMP/PKA pathway, along with transferrin through unknown pathway, contribute to spermatogonia differentiation. FSH ensures entry into meiosis through regulating DNA synthesis and chromosome dynamics. FSH regulates DNA synthesis via temporally inhibiting IL-6, Activin A and inducing Inhibin B. Chromosome dynamics is positively regulated by nociception via cAMP/PKA signaling pathway. Also, FSH upregulates Nrg1 and Nrg3 to induce Stra8 expression in spermatocytes. Besides, limiting gem cell apoptosis by inhibiting Gal3 and Bok is another task of FSH signaling in SCs. Dotted line represents unknown mechanism

Residing in the basement area of the seminiferous tubule, spermatogonia stem cells function as the original sources for the final spermatozoa [148]. In rodents, undifferentiated type A spermatogonia are classified as SSCs and subsequent progenitors [149]. Differentiating spermatogonia are classified into A_1_, A_2_, A_3_, A_4_, intermediate and type B spermatogonia [150, 151]. In humans, undifferentiated spermatogonia are categorized into A_pale_ and A_dark_ spermatogonia. Type B spermatogonia are the differentiating cells [152]. Decades of studies have provided insights into the function of FSH signaling in the spermatogonia pool (Fig. 3).

#### Spermatogonia pool maintenance

Maintaining the spermatogonia pool ensures normal spermatogonia stem cell self-renewal and proliferation of undifferentiated progenitors. FSH has been shown to positively regulate spermatogonia pool maintenance in vivo and in vitro [153–155]. Impaired FSH signaling in immature SCs or mature SCs decreases the colonization of SSCs [156].

Molecular mechanism behind FSH regulation has been elucidated (Table 1). Glial cell line-derived neurotrophic factor (GDNF) and fibroblast growth factor 2 (FGF2) are two factors that are secreted by SCs and positively regulate SSC self-renewal and undifferentiated spermatogonia proliferation [157]. Among them, GDNF activates Akt and MAP kinse-ERK kinase (MEK) signaling pathway, resulting in the production of reactive oxygen species in SSCs. Reactive oxygen species stimulate SSCs self-renewal via the 38 kDa protein (p38) pathway [104, 105]. FSH inhibits autophagy of GDNF in goat SCs via activating the PI3K/Akt/mTOR pathway, which inhibits the translocation of transcription factor EB (TFEB) into the nucleus. Otherwise TFEB induces the expression of lysosomal biogenesis-related genes to degrade GDNF in goat SCs [158]. Recent study reported that GDNF receptor, GDNF family receptor α1, which is expressed by undifferentiated spermatogonia, is also positively regulated by FSH signaling in prepubertal trout testis, though the mechanism is unknown [159]. FGF2 is considered to be a bifunctional factor. For one thing, FGF2 promotes SSC self-renewal along with GDNF [106]. For another thing, FGF2 creates a more suitable environment for SSC differentiation by suppressing GDNF and cytochrome P450 family 26 subfamily B member 1 (Cyp26b1) expression [107]. Possible explanation may be that the combination of GDNF and FGF2 prepares the environment for the formation of progenitors that are ready to differentiate under FSH stimulation. In vivo and in vitro studies using bovine testis and rat testis demonstrates that FSH activates a cAMP-dependent signaling pathway to increase the mRNA levels and protein levels of these two factors [160, 161]. In zebrafish, FSH negatively regulates prostaglandin E2 (PGE2) in SCs by inhibiting Anti Mullerian Hormone (AMH). Otherwise PGE2 promotes SSC self-renewal and inhibits SSC differentiation [108].

#### Spermatogonia differentiation

Undifferentiated type A spermatogonia are under regulation of signaling network to differentiate into differentiated type B spermatogonia and then preleptotene spermatocytes [162]. FSH seems to initiate type A spermatogonia differentiation and induce differentiating spermatogonia proliferation.

Molecular mechanism behind FSH regulation has been elucidated (Table 1). FSH activates RA signaling by increasing RDH10, ALDH1A1, CRABP2 levels in primate SCs and this will provide an environment for induction of spermatogonia differentiation [1]. Stem cell factor (SCF) and steel factor (SLF) are two factors secreted by Sertoli cells during postnatal stages that are essential for the expansion of differentiating spermatogonia [111, 160]. FSH signaling induces transcription of SCF and SLF in prepubertal mouse testis via the cAMP-dependent signaling pathway [112]. The same phenomenon was also observed in adult rat testes [111, 160]. Both SCF and SLF are v-Kit Hardy-Zuckerman 4 Feline Sarcoma Viral Oncogene Homolog (kit) ligands (pleiotropic growth factor) and bind to kit (kit ligands receptor, CD117 is the cluster number for KIT receptor tyrosine kinase) on the surface of differentiating spermatogonia [163]. Owning to alternative splicing, kit ligand has transmembrane form or soluble form at different developmental stages and soluble form is favored for SSC differentiation [112]. Supporting this, transmembrane form of kit ligand is detected immunohistochemically in stages VII-VIII of the mouse seminiferous epithelium, during which SCs are less-sensitive to FSH signaling in mice [111, 164, 165]. Bone morphogenetic protein-4 (BMP4), secreted by SC during early postnatal stage, is proposed to promote SSC differentiation after binding to its receptor on spermatogonia [109, 110]. Its expression is under FSH/cAMP regulation. cAMP analogues downregulate BMP4 expression in prepubertal and pubertal mouse SCs while RA upregulates BMP4 expression level in prepubertal and pubertal mouse SCs [166, 167]. In zebrafish, FSH activates insulin growth factor 3 (Igf3) production via cAMP/PKA pathway [113]. Igf3 promotes SSC differentiation via beta-catenin (β-catenin) pathway in SSCs [114]. Other factors that are regulated by FSH and promote SSC differentiation include transferrin and Doublesex (sex determination and differentiation gene) and mab-3 (sex determination and differentiation gene) related transcription factor [161]. Transferrin functions as an ion transport to provide necessary ions for differentiating spermatogonia [115, 116].

#### Spermatogonia survival

FSH has been shown to protect spermatogonia from apoptosis which is important for steadiness of spermatogonia pool. Bcl-2-related ovarian killer (Bok) is proapoptotic member of the Bcl-2 gene family. FSH downregulates Bok mRNA level in rat testes to inhibit apoptosis [117]. Supporting this result, FSH suppression in immature rat SCs activates the caspase 9 mediated intrinsic apoptotic pathway [118]. Activation of the intrinsic apoptotic pathway is partially attributed to the activation of Bcl-2 gene family [168].

To sum up, FSH signaling in SCs induces paracrine action to maximize the capacity of spermatogonia ecology by maintaining the undifferentiated spermatogonia pool, promoting spermatogonia differentiation and spermatogonia survival. How to achieve a balance between molecules promoting self-renewal and molecules promoting differentiation in response to FSH stimulation remains to be further investigated. The answer may reside in spermatogonia themselves because it was reported that germ cells can control the local balance of GDNF, BMP4 and kit ligand levels [161].

### Entry into meiosis and spermatocyte survival

Transition of type B spermatogonia into spermatocytes facilitates meiosis while surviving spermatocytes are essential for quantitative spermatogenesis. FSH is shown to be indispensable for entry into meiosis and positively regulates spermatocyte survival (Fig. 3).

#### Entry into meiosis

In vitro study using coculture system containing SCs and spermatogonia showed that FSH initiates the differentiation of secondary spermatogonia into primary spermatocytes in newts [169]. Injection of *hpg* mice with exogeneous FSH or transgenic expression of FSH restores the number of spermatogonia and spermatocytes [170, 171]. This observation is supported by hypophysectomised or GnRH-immunized adult rat models which lack normal circulating FSH levels [26, 172]. Furthermore, knocking out of FSHR and FSHβ in mice resulted in decreased numbers of spermatogonia and spermatocytes, let alone spermatids [25, 173]. These results indicate that FSH is necessary for spermatogonia to differentiate into primary spermatocytes, promoting entry into meiosis.

Detailed mechanisms that FSH adopts to guarantee entry into meiosis are as follows (Table 1). Activin alpha (Activin A, growth and differentiation gene) and Inhibin beta (Inhibin B, growth and differentiation gene) are two structurally-related factors that belong to the transforming growth factor β family. Activin A promotes DNA synthesis in spermatocytes while Inhibin B inhibits this biological process [119]. Through the cAMP/PKA signaling pathway, FSH can activate the production of Inhibin B while inhibit the production of Activin A near the beginning of meiosis [120]. Thus, FSH functions as a monitor for the end of DNA synthesis. This result is further supported by a study of another factor interleukin 6 (IL-6). IL-6 is reported to negatively regulate DNA synthesis. IL-6 expression is downregulated by FSH via the cAMP dependent pathway during stages VII-VIII and upregulated by FSH via the PKC dependent pathway during stages IX-XI in rats [121]. Stages VII-VIII corelate with the initiation of meiotic DNA synthesis while stages IX-XI correlate with DNA synthesis termination. Recently, Eto et al. reported that FSH can promote nociception expression via cAMP/PKA signaling in murine Sertoli cells [122]. Nociceptin (17-residue neuropeptide) is secreted by Sertoli cells and binds to nociception receptor opioid related nociceptin receptor 1 (OPRL1) on the surface of spermatocytes [123]. Binding of nociception to its receptor leads to REC8 meiotic recombination protein (Rec8) phosphorylation in spermatocytes which promotes meiotic chromosome dynamics to prepare for the subsequent meiosis [174]. Similarly, FSH, combined with retinoic acid, stimulates Neuregulin 1 (Nrg1) and Neuregulin 3 (Nrg3) expression in mouse SCs [124, 125]. Nrg1 and Nrg3 are secreted from SCs and bind to their receptor EGFR – Mouse Genome Informatics 4 (ERBB4) on the surface of pre-spermatocytes which will trigger stimulated retinoic acid gene 8 (Stra8) expression [125]. Upregulated Stra8 expression promotes the early stage of meiotic prophase [175]. However, the exact signaling pathway that is adopted by FSH remains to be elucidated. In summary, these results demonstrates that FSH positively regulates entry into meiosis by temporally ensuring the initiation of DNA synthesis and termination of DNA synthesis, as well as monitoring meiotic chromosome dynamics.

#### Spermatocyte survival

FSH is also pivotal for spermatocyte survival. In FSH-suppressed adult rats and gonadotropin-suppressed adult men, the spermatocyte apoptosis rate showed a significant increase [176, 177].When the androgen level is normal, the suppression of FSH reduced pachytene spermatocytes numbers in rats [178]. In human SCs, Sá et al. found that the combination of FSH and testosterone maximally maintains spermatocytes because FSH alone was not enough to limit spermatocytes apoptosis during the second week of vero cell conditioned medium [179, 180]. Previous study demonstrated that spermatocyte apoptosis is related to both extrinsic (Caspase 8) and intrinsic (Caspase 9) apoptotic pathways [181, 182]. Supporting this finding, FSH signaling in rats has been shown to inhibit both the extrinsic and intrinsic apoptotic pathways during the first wave of spermatogenesis to promote spermatocyte survival [118].

Another factor, Galectin-3 (Gal-3) is reported to inhibit both the intrinsic and extrinsic apoptotic pathways by blocking cytochrome c release and Fas (death receptor)/Fas-ligand (member of the tumor necrosis factor family of death-inducing ligands)cross linking respectively [126]. FSH induces expression of Gal-3 in porcine and rat SCs at the initiation stage of meiosis and protects spermatocytes from apoptosis, probably via cAMP/PKA dependent pathway [127].

Additionally, FSH can inhibit early meiotic spermatocyte apoptosis via inhibition of transcription factor AP-1 in human SCs. Activation of AP-1 occurs before the activation of effector caspase such as caspase 3 [128]. Caspase 3 was shown to be expressed in human SCs and germ cells [183]. This indicates that FSH signaling in human SCs may control germ cell death via paracrine action.

In summary, FSH exerts its effect at the beginning of meiosis by promoting entry into meiosis and the survival of spermatocytes. This effect may be mainly due to the sufficient number of Sertoli cells and spermatogonia. Further studies are recommended to focus on the effect of FSH on the transition from spermatogonia to spermatocytes as well as whether FSH has effect on the transition from primary spermatocytes to the secondary spermatocytes.

## Potential use of follicle-stimulating hormone in treating male infertility

In humans, FSH induces SC proliferation and spermatogonia proliferation and differentiation, while FSH alone is not essential to complete meiosis and spermiogenesis [184, 185]. Testosterone is more important from the beginning of spermatocyte development [186]. The differences in the functions of FSH signaling in spermatogenesis between humans and other experimental animals suggest that further studies should be conducted to understand the FSH regulation in human or we should develop more appropriate experimental animal models.

Currently, FSH treatment is mainly administrated to two types of patients: patients with hypogonadotropic hypogonadism (HH) and normogonadotropic patients with idiopathic impairment of spermatogenesis [187]. The findings described above indicate that FSH promotes the final sperm production by positively regulating Sertoli cell biology. As expected, questions about whether excess FSH is harmful for spermatogenesis arise before FSH treatment. In rodents, high serum FSH levels result in better testis development [78, 188]. Men with pituitary adenoma secreting excess FSH also show normal spermatogenesis and normal testicular development [189]. Supporting this result, enhanced receptor activity resulted from gain of function mutations in FSHR, such as FSHR-D567G and FSHR-N431I, also appears to have little effect on normal spermatogenesis [190, 191]. These results provide a theoretical support for FSH treatment.

In patients with hypogonadotropic hypogonadism, the lack of gonadotropin FSH stimulation or defects in gonadotropin-releasing hormone synthesis and secretion lead to azoospermia or severe oligozoospermia [187, 192]. One method to treat HH is pulsatile GnRH administration which may lead to the secretion of gonadotropin from the pituitary gland [29, 193]. The secreted FSH can stimulate Sertoli cell growth to support normal spermatogenesis. However, this method is costly and troublesome since external GnRH must be pumped subcutaneously [194]. Another method to treat HH is the administration of exogenous gonadotropins. This method, which involves treating patients with human chorionic gonadotropin alone or in combination with FSH, is more direct and may be more successful in most cases [195, 196]. Human chorionic gonadotropin (hCG) functions similarly to luteinizing hormone, but with different bioactivities [197]. hCG is observed to restore sperm production in men. This effect may be enhanced when hCG is administered in combination with FSH [198, 199]. However, the precise dosage and timing of FSH treatment in this method remains controversial. Though this method seems useful at present, limitations have also been noted. For example, exogenous gonadotropin administration is not the same as the gonadotropin secretion stimulated by GnRH. The intrinsic regulatory network cannot be simulated using this method.

FSH treatment appears to be beneficial for normogonadotropic patients with idiopathic impairment of spermatogenesis. Meta-analyses revealed that FSH therapy in these patients increases the rate of clinical pregnancies in female partners [200]. However, since the number of participants taking part in the experiment was relatively low, studies are still needed to determine whether FSH therapy truly affects normogonadotropic patients. As a result, the selection of appropriate normogonadotropic patients to receive FSH therapy is necessary. First, no identifiable and generally accepted cause for male infertility should be detected [201]. Second, FSH pharmacogenetics is promising in this evaluation. Testing for the single nucleic polymorphism (SNP) p.N680S in patients receiving FSH therapy is important. Male patients with the p.N680S homozygous N polymorphism exhibit a significantly decreased DNA fragmentation index of sperm in the ejaculate after FSH treatment [202–204]. Also, SNP of the FSHβ is another marker to select the normogonadotropic patients to receive FSH therapy [205, 206]. More well-organized and sufficient randomized studies are needed to determine whether FSH therapy is truly helpful for normogonadotropic patients as well as the dosage and timing needed to carry out therapy. In summary, Precision Medicine matters a lot!

Let’s return to the exogeneous gonadotropin administration method. How to expand the half-life of gonadotropin is important for treatment efficiency. One way is to conjugate the gonadotropin to polyethylene glycol (PEG). PEGylated FSH not only retains FSH activity but also results in improved bioavailability [207]. Another way is to develop a single-chain recombinant analogue of gonadotropin [208]. These molecules were engineered with the β-subunits oriented at the N-terminus of the α subunit and used the hCGβ carboxy-terminal peptide (CTP) sequence as a linker [209, 210]. These analogues have an increased serum half-life and increased biopotency. Using this method, we obtain dual FSH and LH analogues, such as FSHβ-CTP-LHβ-CTP-α [211]. Recently, fusion analogues of FSH consisting FSHα, FSHβ and immunoglobulin constant fragments were constructed [212]. This type of analogue can improve pharmacokinetics. All the aforementioned analogues have great beneficial to female infertility treatment and ovarian development of experimental animals such as sheep and monkeys. It remains to be determined if present analogues can treat male infertility. Analogs that can be used in clinical trials are being researched [31].

Recently, the relationship between diabetes mellitus and male infertility attracts attention [213–215]. As the metabolic modulator in seminiferous tubule, SC metabolism dysfunction is thought to be one link between diabetes mellitus and male infertility [216, 217]. In human, Glut1 and Glut3 transport glucose into SCs. With the help of lactate dehydrogenase (LDH), glucose can be converted into lactate which will be transported out of SCs and supplied to germ cells via monocarboxylate transporter (MCT) [218–220]. Diabetes mellitus patients displayed low level of FSH, low mRNA levels of Glut1, Glut3 and low protein level of LDH in SCs [221]. Insulin-deprived human SCs, which was similar to diabetes mellitus, presented decreased transcript level of LDH, MCT4, Glut3 and increased transcript level of Glut1 [222]. Moreover, decreased level of sirtuin 1 and increased level of ghrelin in diabetes mellitus patients impair the hypothalamus-pituitary–gonadal axis which leads to low level of FSH [86, 223, 224]. In Klinefelter syndrome male patients which is prone to suffer from diabetes mellitus, high level of FSH along with increased mRNA expression of Glut3 and decreased mRNA expression of Glut1 in SCs may be a try to rescue spermatogenesis [225]. Based on this, drugs can be developed to rescue FSH in diabetes mellitus patients so that normal SC metabolism can occur and sufficient energy can be provided to germ cells.

Hope still exists. Conversation in spermatogenic processes between human and mouse are revealed in previous studies [226]. Since the phenotypes of *Fshr*-knockout mice and men carrying *Fshr* mutations are similar, the *Fshr*-knockout mouse model still has great clinical potential [29]. The identification of additional genes that are regulated by FSH in mice and developing targeted medicines are feasible. Last, the combination of FSH and testosterone treatment is more efficient than a single hormone treatment, since testosterone can augment FSH signaling in SCs [227].

## Conclusion

FSH signaling in SCs establishes the appropriate microenvironment for spermatogenesis. FSH signaling plays a dominant role in determining the number and function of SCs. FSH signaling also maintains the spermatogonia pool and induces spermatogonia differentiation through paracrine actions. In addition, FSH signaling promotes entry into meiosis and the survival of germ cells. However, few molecules involved in these paracrine actions have been found. We could detect the changes within protein and mRNA expression level of receptors on the surface of gem cells that are associated with different spermatogenic processes, and then determine whether the levels of their ligands changed after the administration of FSH signaling in SCs. Mass spectrometry and single-cell transcriptomics will be helpful. Moreover, combining the transgenic mouse model with human infertility is necessary to develop therapies for diseases related to dysfunctional FSH signaling. 

## Data Availability

All data generated or analyzed during this study are included in this published article.

## Abbreviations

ɑ6β1 integrin: Alpha6beta1-integrin
β-catenin: Beta-catenin
AC: Adenylate cyclase
Activin alpha: Activin A
AEA: N-arachidonoylethanolamine
Akt: Protein kinase B
ALDH1A1: Aldehyde dehydrogenase 1A1
AMH: Anti Mullerian Hormone
AP-1: Activator protein-1
Aqp8: Aquaporin 8
ASNS: Asp synthetase
BMP4: Bone morphogenetic protein-4
Bok: Bcl-2-related ovarian killer
BTB: Blood-testis barrier
cAMP: Cyclic adenosine monophosphate
c-Myc: Cell-derived Myc
c-jun: Cell-derived jun proto-oncogene
CRABP2: Cytoplasmic RA-binding protein 2
CREB: Cyclic AMP response-element binding protein
CTP: Carboxy-terminal peptide
Cyclin D1: Type D1 cyclin
Cyp26b1: Cytochrome P450 family 26 subfamily B member 1
DAX1: Nuclear receptor subfamily 0 group B member 1
ERBB4: EGFR – Mouse Genome Informatics 4
ERK: Extracellular-regulated kinase
ERK1/2: Extracellular signal-regulated protein kinases 1 and 2
FAAH: N-arachidonoylethanolamine hydrolase
FGF2: Fibroblast growth factor 2
FSH: Follicle-stimulating hormone
FSHR: Follicle-stimulating hormone receptor
G proteins: Heterotrimeric guanine nucleotide–binding proteins
Gal-3: Galectin-3
GDNF: Glial cell line-derived neurotrophic factor
GnRH: Gonadotropin-releasing hormone
hCG: Human chorionic gonadotropin
HH: Hypogonadotropic hypogonadism
HIF: Hypoxia inducible factor
hpg: Hypogonadal
Hippo: Ste20-like protein kinase Hippo
Igf3: Insulin growth factor 3
IL-6: Interleukin 6
Inhibin B: Inhibin beta
Kit: V-Kit Hardy-Zuckerman 4 Feline Sarcoma Viral Oncogene Homolog
Klf4: Krüppel-like factor 4
LDH: Lactate dehydrogenase
LH: Luteinizing hormone
MAPK: Mitogen-activated protein kinase
MCT: Monocarboxylate transporter
MEK: MAP kinase-ERK kinase
mTOR: Mammalian/mechanistic target of rapamycin
mTORC1: Mammalian target of rapamycin complex 1
N-cadherin: Neural-cadherin
NF: Nuclear factor
Nrg1: Neuregulin 1
Nrg3: Neuregulin 3
OCT4: Octamer-binding transcription factor 4
p38: 38 KDa protein
OPRL1: Opioid related nociceptin receptor 1
p70S6K: Phosphorylated 70-kDa ribosomal S6 kinase
PDK3: Pyruvate dehydrogenasekinase 3
PEG: Polyethylene glycol
PFKFB: 6-Phosphofructo-2-kinase/fructose-2,6-biphosphatase
PFKFB1: 6-Phosphofructo-2-kinase/fructose-2,6-biphosphatase isoform 1
PFKFB3: 6-Phosphofructo-2-kinase/fructose-2,6-biphosphatase isoform 3
PGE2: Prostaglandin E2
PI3K: Phosphoinositide 3-kinase
PIP2: Phosphatidylinositol bisphosphate
PIP3: Phosphatidylinositol 3,4,5-trisphosphate
PKA: Protein kinase A
PRAS40: Proline-rich Akt substrate of 40 kDa
RA: Retinoic acid
RARE: RA response element
RARɑ: Retinoic acid receptor ɑ
RDH10: Retinol dehydrogenase 10
Rec8: REC8 meiotic recombination protein
RXR: Retinoid X receptor
SC: Sertoli cell
SCF: Stem cell factor
SLF: Steel factor
SNP: Single nucleic polymorphism
SSC: Spermatogonia stem cell
Stra8: Stimulated retinoic acid gene 8
TFEB: Transcription factor EB
tPA: Tissue plasminogen activator
Wnt3: Wingless-type MMTV integration site family, member 3
YAP: Yes-associated protein
